# Next-generation sequencing guides diagnosis and treatment in a complex presentation of ALK-positive anaplastic large-cell lymphoma: a case report

**DOI:** 10.3389/fonc.2025.1502782

**Published:** 2025-03-14

**Authors:** Tejasvini Vaid, Thomas S. Gunning, Rachel Cohen, Alexandra Della Pia, Jason Voss, Melinda Weber, Andrew L. Pecora, Lori A. Leslie, Tatyana Feldman, Andre H. Goy, Maher Albitar, Andrew Ip

**Affiliations:** ^1^ Department of Medical Oncology and Hematology, Medanta-The Medicity, Gurgaon, India; ^2^ Department of Oncology, Hackensack Meridian School of Medicine, Nutley, NJ, United States; ^3^ American Medical Program, Tel Aviv University, Tel Aviv, Israel; ^4^ John Theurer Cancer Center, Hackensack Meridian Health, Hackensack, NJ, United States; ^5^ Genomic Testing Cooperative, Irvine, CA, United States

**Keywords:** lymphoma, NGS, next-generation sequencing, targeted therapy, case report

## Abstract

Next-generation sequencing (NGS) technology is being increasingly utilized in the management of cancer patients due to its diagnostic, therapeutic, and prognostic value, and potential to inform use of targeted therapy. We report a case wherein performing NGS testing proved to be a critical component in diagnosis and therapeutic decision making. The case was of a patient who presented with diffuse osteolytic bone lesions that on biopsy showed an undifferentiated malignancy. A diagnosis of poorly differentiated sarcoma was made at an outside institution and carboplatin and paclitaxel was initiated. However, NGS testing revealed a *TRAF1::ALK* translocation, which led to a revised diagnosis of stage IV *ALK*-positive anaplastic large cell lymphoma (ALCL), a curable cancer. The patient then started treatment with brentuximab vedotin, cyclophosphamide, doxorubicin, etoposide, and prednisone followed by autologous stem cell transplantation consolidation, given the very extensive disease at presentation. She remains in continued complete remission at 28 months. In this case, NGS was essential in establishing the correct diagnosis and selection of therapy in high-risk ALCL. NGS testing should be a routine component of the oncology patient workup to complement standard diagnostic modalities.

## 1 Introduction

The utilization of next-generation sequencing (NGS) technology to identify molecular signatures has transformed the diagnosis and management of cancer patients (1). Historically, the workup for malignancies included histopathological diagnosis, genetic studies such as cytogenetics and fluorescence *in-situ* hybridization (FISH), disease staging using imaging, and prognostic evaluation via clinical indices. Progress in cancer cell biology has shed light on the impressive molecular diversity of cancer, in particular since the human genome sequencing and parallel acceleration of high throughput technology in molecular testing well beyond targeted sequencing. This has led to the advent of genomics with targeted NGS currently accessible in routine practice, with the potential to impact all phases of cancer care from refining diagnosis, helping define therapeutic choices, and prognostication/stratification of patients.

Precision diagnosis using NGS has led to the further subclassification of specific malignancies into distinct molecular subtypes. In the case of malignancies that have been historically challenging to accurately diagnose, such as T-cell lymphomas, NGS has clarified disease classification and allowed us to distinguish between nuanced entities (2). Furthermore, NGS may be a valuable tool when the histopathological diagnosis is uncertain. In several cases, the identification of molecular subtypes by NGS goes beyond just a diagnostic purpose; it has well-defined therapeutic and prognostic implications as well (3, 4). A comprehensive understanding of the molecular mutations and downstream pathways at play allows for the administration of novel targeted therapies and the potential to perform minimal residual disease assessments, which may greatly increase the chance of treatment success and optimize patient outcomes (5).

The therapeutic utility of NGS can extend beyond the detection of a single targetable mutation. It can provide insight into the complex mutational landscape of the tumor such as the presence of synergistic mutations, mutations that confer resistance to targeted therapies, and even mutations associated with a high incidence of relapse (6). This makes NGS a useful tool in the armamentarium for not only accurate prognosis and diagnosis, but also early recurrence detection.

**Figure 1:**
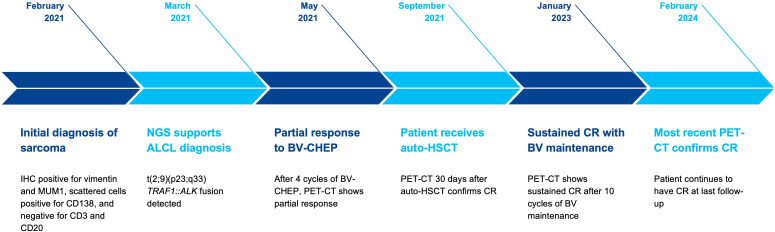
Schematic illustrating the patient’s timeline from diagnosis with next-generation sequencing leading to treatment with complete response for ALK-positive anaplastic large-cell lymphoma.

Here, we describe a case wherein performing NGS testing proved to be crucial in the appropriate diagnosis and treatment of a patient with anaplastic large cell lymphoma (ALCL) (**Figure 1**).

## 2 Case presentation

A 33-year-old female presented with a complaint of fever and diffuse bone pain for approximately one month in early 2021. She reported previous concerns with her knees, explaining sudden weakness which led her to fall. Beside asthma and a history of anemia with each of her two prior pregnancies, she reported no prior health concerns. She was admitted to an outside hospital for evaluation of her generalized fatigue and pain. Computed tomography (CT) imaging revealed diffuse osteolytic bone lesions and laboratory testing reported hypercalcemia (17 mg/dL). Complete blood count testing showed a slight leukocytosis (13.7 K/μL) and anemia (hemoglobin 9.2 g/dL). The trilineage hematopoiesis on bone marrow aspirate smear showed a few scattered large cells (1%-2% of total cells) with a moderate amount of basophilic cytoplasm and abundant cytoplasmic vacuoles. The lineage of these atypical cells was unclear, but minimal marrow involvement by a neoplastic process (including lymphoma or multiple myeloma) could not be excluded. On evaluation at our hospital, initial workup via interventional radiology-guided biopsy of a bone lesion revealed proliferation of discohesive, highly anaplastic plasmacytoid tumor cells. Mitotic activity was very high and Ki-67 proliferation index was >90%. Immunostains showed that tumor cells were positive for vimentin and MUM1, and scattered tumor cells were positive for CD138 and CD163. It was presumed that the patient had a poorly differentiated sarcoma and she was subsequently referred to our cancer center for treatment. Given the extent of her disease, she was initially treated with carboplatin and paclitaxel while awaiting NGS results. Despite treatment, the patient did not experience improvement in her pain and was subsequently wheelchair bound.

**Figure 2:**
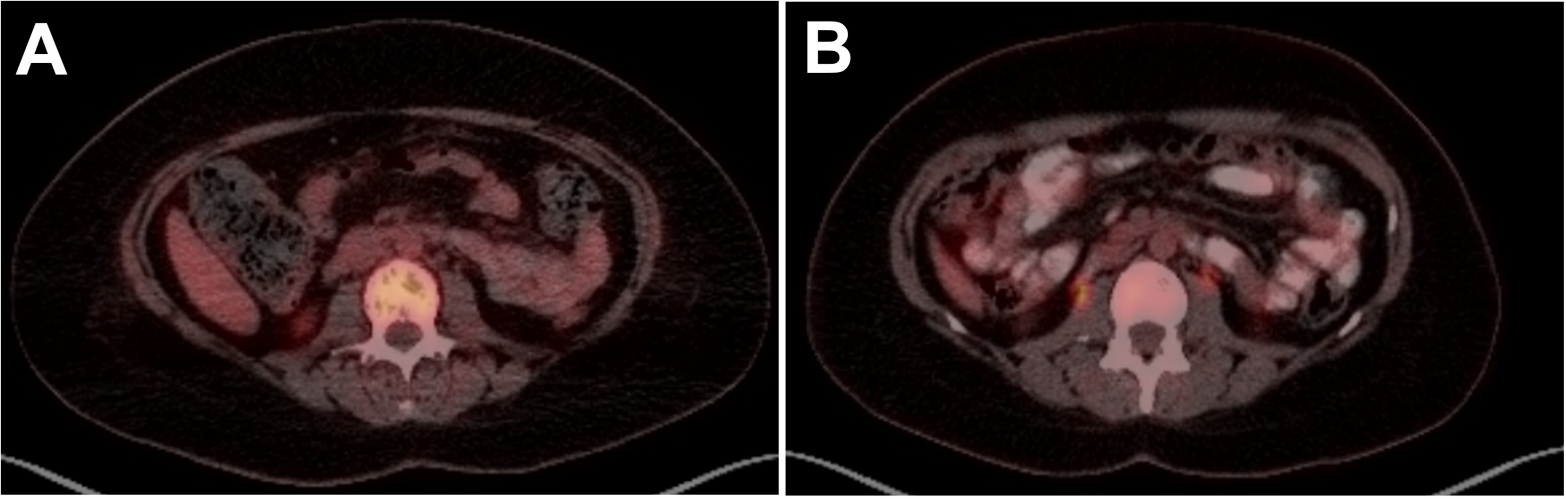
PET scan images of lumbar spine in a 33-year-old female with ALK-positive anaplastic large cell lymphoma (ALCL) at **(A)** diagnosis showing widespread osteolytic lesions and mottled bones and **(B)** 16 months post-HSCT showing CR with resolution of osteolytic lesions and mottled bone.

Following initial treatment, further work-up included NGS testing using targeted DNA and RNA sequencing based on hybrid capture technology. DNA sequencing included all coding exons of 177 genes and targeted RNA of 1408 genes for the purpose of quantifying the expression levels as well as the detection of various fusions that may involve any of the 1408 genes. NGS testing revealed a *TRAF1::ALK* and elevated expression levels for Ki-67, ALK, IL2RA, and PD-L1 were also identified (**Table 1**). She was subsequently referred to the lymphoma division, where additional work-up included flow-cytometry, molecular testing, and further immunohistochemistry (**Table 1**). These stains showed CD30 and CD4 positivity and confirmed *ALK*-positive ALCL. At 1 month from her initial presentation, a positron emission tomography (PET)-CT showed widespread osteolytic lesions and mottled bones with diffuse marrow uptake (**Figure 2A**).

**Table 1:** Results summary of pathological and genomic testing for the diagnosis of this case of *ALK*-positive anaplastic large-cell lymphoma.

Testing	Result
Next-generation sequencing	
t(2;9)(p23;q33) *TRAF1::ALK* fusion	Detected
Structural and numerical chromosomal abnormalities	-3p, +7q, +17q, and -20p
* ALK* expression	High
* IL2RA* expression	High
* Ki-67* expression	High
* PD-L1* expression	High
Flow Cytometry	
CD45	Bright
CD2	Moderate
CD3	Moderate
CD4	Moderate
CD5	Moderate
CD7	Moderate
CD8	Bright
Molecular testing	
c-*myc* translocation FISH	Negative
* BRAF* mutation assay	Negative
* NRAS* mutation assay	Negative
c-*KIT* mutation assay	Negative
Immunohistochemistry	
*ALK* (cytoplasmic)	Positive
CD30	Positive
CD4	Positive
CD43	Positive
CD3	Negative
CD5	Negative
CD20	Negative

The diagnosis was confirmed as stage IV *ALK*-positive ALCL and she started on treatment with brentuximab vedotin, cyclophosphamide, doxorubicin, etoposide, and prednisone (BV-CHEP). PET-CT was performed after 2 cycles of therapy, but her response was difficult to interpret due to stable extensive lytic osseous disease. A repeat PET-CT after Cycle 4 confirmed the improvement and was read as a partial response (PR). The patient completed 6 cycles of BV-CHEP with minimal complications including abdominal pain and neutropenic fever throughout her treatment course. At 5 months following her initial presentation and completion of treatment, PET-CT showed no hypermetabolism in neck/chest/abdomen/pelvis, mixed lytic-blastic process in bony structures, and less pronounced heterogenous hypermetabolism. Given these findings and the extent of disease at presentation, it was decided to proceed with high-dose therapy and consolidation with autologous hematopoietic stem cell transplantation (auto-HSCT). The patient proceeded to ifosfamide and etoposide stem cell mobilization and collection, followed by conditioning with carmustine, etoposide, cytarabine, and melphalan, and auto-HSCT 7 months after initial presentation. At 30 days following auto-HSCT, PET-CT showed a complete resolution of all findings and confirmed a complete response (CR). The patient continued on brentuximab vedotin maintenance therapy for 10 cycles. She has remained in CR with no evidence of disease observed in 2 PET-CT imaging scans at 24 months and 37 months, her last routine follow-up visit (**Figure 2B**).

## 3 Discussion

NGS has revolutionized DNA sequencing in order to improve diagnostic accuracy and therapy through its efficient, high-throughput parallel sequencing. This is especially important when it comes to cancer and clonal evolution. Therefore, as demonstrated in our case, NGS can help improve diagnostic accuracy, allowing for appropriate treatment.

In this patient, the original diagnosis had been reviewed at an outside institution, with highly aggressive and undifferentiated histology on the bone marrow biopsy and on the targeted bone lesion by interventional radiology. Pathologic testing could not exclude marrow involvement by lymphoma or multiple myeloma. However, the initial lymphoma panel conducted via IHC for this patient was limited to CD3 and CD20 only, and negative results for both reduced suspicion of lymphoma and prompted cessation of further lymphoma workup. This case highlights the importance of thorough morphologic assessment and complete IHC workup, as these would have helped reach the correct diagnosis. Crucially, here the diagnosis was redirected towards a T-cell lymphoma via NGS testing, which identified a *TRAF1::ALK* translocation. Additional IHC testing was strongly positive for ALK1 (cytoplasmic), CD30, and CD4, corroborating the NGS results. These findings (**Table 1**) eventually led to the correct diagnosis of *ALK*-positive ALCL. Despite the availability of expert pathologists and state-of-the-art diagnostic tools, the pleomorphic histology associated with T-cell lymphomas can obfuscate diagnosis. In such cases, performing NGS during the initial evaluation helps confirm the oncogenic abnormalities and avoid misdiagnosis (7, 8). A study by Bommier et al. revealed that NGS was helpful in correctly diagnosing 74% of angioimmunoblastic T-cell lymphoma cases (diagnosis was confirmed in 69.6% of cases and changed in 4.4% of cases), where the histological diagnosis was “not sure” in 61% of cases (7).

Similarly, in the case of B-cell lymphomas, the available data support the use of NGS in suspected cases of CD10^+^ B-cell primary central nervous system lymphomas to avoid diagnostic errors (9, 10). Diagnostic confirmation was achieved in 100% of the patients with primary central nervous system lymphomas through NGS, while the histological diagnosis was “moderately sure” in only 42% cases (7). NGS has also refined the classification of diffuse large B-cell lymphoma molecular subtypes and delineated subtypes with distinct prognostic impact. For example, diffuse large B-cell lymphoma with *SOCS1* mutation–which can be identified through NGS–has a better 5-year overall survival after treatment with rituximab, cyclophosphamide, doxorubicin, vincristine, and prednisone as compared to other molecular subtypes (11).

NGS is especially useful in the diagnosis of lymphomas that have an unambiguous mutational landscape. The *TRAF1::ALK* translocation detected in our patient is a rare genetic abnormality that has a distinct prognostic implication. Unlike the conventional *NPM-ALK* translocation, the *TRAF1::ALK* translocation in ALCL is associated with a high incidence of relapse (12). The detection of this translocation on NGS was a deciding factor for performing a consolidative autologous stem cell transplantation during first remission. A similar case of ALCL was described by Agarwal et al, who also performed an upfront auto-HSCT following NGS-based identification of this aggressive translocation (13). While FISH testing could potentially detect the rearrangement, the role of FISH in ALCL workup remains confirmatory and prognostic, rather than a modality for diagnosis (14). Still, FISH versus NGS testing for ALK rearrangements is an ongoing area of debate, as the type of rearrangement may affect the accuracy of FISH in lung cancers, for example (15). Even so, FISH analysis in the evaluation of lymphomas focuses on common rearrangements, such as c-myc, which was performed and negative in this patient’s workup (16).

Through this case, we demonstrate that NGS, especially sequencing both DNA and RNA, is a valuable tool for the diagnosis and treatment of challenging lymphoma cases as well as for advancing our understanding of the biological and oncogenic landscape of lymphomas. Unfortunately, NGS is still not used routinely, due to factors limiting access. The heterogeneity of payers in the United States and inconsistent findings from health technology assessments (eg, cost-effectiveness analyses) that sought to quantify the economic value of NGS have engendered disparate levels of insurance coverage. The predominance of single-arm studies focused on short-term outcomes within the field of oncology is antithetical to traditional economic evaluations, which rely on head-to-head comparisons and long time horizons. Additionally, the range of NGS applications within oncology can prompt hesitancy among payers, who are intent on mitigating misguided healthcare resource utilization through the diagnostic and treatment-determining capacity of NGS, rather than covering its use as a prognostic tool (17, 18).

Gaps in coverage for NGS shunts costs to patients, increasing risk of financial toxicity (19). In an economic study that sought to quantify the cost of mutation testing in patients with metastatic non-small cell lung cancer, investigators employed decision tree modeling to estimate that per patient costs for NGS testing would be $6,225 and $2,099 for commercial and Medicare beneficiaries, respectively. Despite these high costs, NGS was estimated to be less expensive than polymerase chain reaction-based approaches, with NGS providing the added benefit of conferring the fastest time to initiation of appropriate targeted therapy (20). A literature review of studies evaluating the cost-effectiveness of NGS across tumor types concluded that NGS was associated with cost savings versus single-gene testing, agnostic of malignancy. The magnitude of health-economic benefit was enhanced as more genes were evaluated, making NGS a particularly useful modality in cancers characterized by many actionable genomic alterations (21).

A key barrier to NGS is the absence of standardized pharmacoeconomic tools for personalized medicine in oncology, which has fostered incertitude among payers and an obscure coverage landscape. However, the emerging focus on developing health technology assessments that enable NGS decision-making may clarify its standing in cost-conscious, real-world practice (19, 22, 23). A recent analysis of NGS testing rates in select solid tumors before and after the national coverage determination rendered by the Centers for Medicare & Medicaid Services in 2018, which classified NGS as reasonable, necessary, and reimbursable for oncology patients when performed in a Clinical Laboratory Improvement Amendments–certified laboratory, revealed that NGS testing rates have increased in certain tumor types, but not others (24). Additional evidence demonstrating the utility of NGS in a broad variety of cancers, including hematological neoplasms as reviewed in this patient case, can help support broader receptivity and increased use of NGS.

## 4 Conclusions

This case report can serve as an example for how NGS and testing both DNA and RNA can be instrumental in redirecting diagnosis and therapy. Though NGS is more commonly used in certain solid tumor subtypes, there is mounting evidence that this molecular test is of paramount importance in precision medicine to improve patient outcomes. Despite advancements in payer policies to expand coverage of NGS and molecular biomarker–based therapy approvals, NGS rates have remained low across tumor types. Given the potential for improved patient outcomes with molecular biomarker–based therapy, further efforts to improve NGS usage rates are warranted.

## Data Availability

The raw data supporting the conclusions of this article will be made available by the authors, without undue reservation.
